# Characterizing Behavioral Activity Rhythms in Older Adults Using Actigraphy

**DOI:** 10.3390/s20020549

**Published:** 2020-01-19

**Authors:** Ariel B. Neikrug, Ivy Y. Chen, Jake R. Palmer, Susan M. McCurry, Michael Von Korff, Michael Perlis, Michael V. Vitiello

**Affiliations:** 1 Department of Psychiatry and Human Behavior, University of California, Irvine, CA 92697, USA; cheniy1@hs.uci.edu; 2 Department of Psychology, Macquarie University, Sydney, NSW 2113, Australia; jake.palmer@sydney.edu.au; 3 Healthy Brain Ageing Program, Brain and Mind Centre, University of Sydney, Sydney, NSW 2006, Australia; 4 Department of Child, Family, and Population Health Nursing, University of Washington, Seattle, WA 98195, USA; smccurry@uw.edu; 5 Department of Psychiatry and Behavioral Sciences, University of Washington, Seattle, WA 98195, USA; vonkorff.m@gmail.com (M.V.K.); vitiello@uw.edu (M.V.V.); 6 Penn Behavioral Sleep Medicine Program, Department of Psychiatry, University of Pennsylvania, Philadelphia, PA 19104, USA; mperlis@upenn.edu

**Keywords:** actigraphy, circadian rhythms, older adults, behavioral activity rhythms

## Abstract

Wrist actigraphy has been used to assess sleep in older adult populations for nearly half a century. Over the years, the continuous raw activity data derived from actigraphy has been used for the characterization of factors beyond sleep/wake such as physical activity patterns and circadian rhythms. Behavioral activity rhythms (BAR) are useful to describe individual daily behavioral patterns beyond sleep and wake, which represent important and meaningful clinical outcomes. This paper reviews common rhythmometric approaches and summarizes the available data from the use of these different approaches in older adult populations. We further consider a new approach developed in our laboratory designed to provide graphical characterization of BAR for the observed behavioral phenomenon of activity patterns across time. We illustrate the application of this new approach using actigraphy data collected from a well-characterized sample of older adults (age 60+) with osteoarthritis (OA) pain and insomnia. Generalized additive models (GAM) were implemented to fit smoothed nonlinear curves to log-transformed aggregated actigraphy-derived activity measurements. This approach demonstrated an overall strong model fit (R^2^ = 0.82, SD = 0.09) and was able to provide meaningful outcome measures allowing for graphical and parameterized characterization of the observed activity patterns within this sample.

## 1. Introduction

The aging process involves major physiological changes including changes in sleep, circadian rhythms, and daily behavioral patterns [1]. Disrupted patterns are more common and disabling in older adults with chronic illness such as dementia, chronic pain, and cancer, each of which are disorders mechanistically linked to the disruption of the endogenous circadian clock [1,2,3]. It is hypothesized that both deterioration of the hypothalamic suprachiasmatic nucleus (SCN), the endogenous clock of the brain, and its consequent weakened functioning as well as inadequate (weak or missing) external cues that are necessary to entrain the endogenous rhythm-regulating behavioral patterns (e.g., sleep/wake and activity) impact older adults [4]. Circadian rhythms are biologically characterized by diverse biomarkers, most commonly, melatonin. However, the procedures involved in measuring such biomarkers are costly, cumbersome, and unrealistic in larger clinical trials, especially those involving older adults. Researchers have considered different approaches to estimate endogenous circadian rhythms, mainly through the use of accelerometry devices known as actigraphs [5]. While other methodologies exist for the assessment of sleep and sleep/wake patterns, here we focus on actigraphy as it is used for evaluation of rhythms [6,7].

Actigraph devices were originally utilized for the evaluation of sleep variables such as total sleep time, time in bed, and sleep efficiency [8,9], and multiple guidelines and reviews considering the validity and reliability of actigraph devices in the study of sleep have been published [10,11,12,13,14,15,16,17,18,19,20]. When compared to overnight polysomnography, the gold standard assessment of sleep, actigraphs are reported to have as high as 97% sensitivity but as low as 24% specificity [18]. Generally, the actigraph shows high sensitivity for detection of wakefulness, especially during the sleep period. However, sensitivity is highly dependent on device used, the algorithm applied, and the type of population with more clinically complex populations showing lower sensitivity. Over the years, other applications of the actigraph emerged, including the assessment of physical activity [21] and circadian activity rhythms [22,23]. The actigraph, a lightweight, compact, and wearable technology, is designed to record movement activity over time. Movements are detected by accelerometers (or piezoelectric accelerometers) that sense accelerations resulting from body movements [24]. While various devices are commercially and generically referred to as actigraphs, in this review, we limit the term “actigraphs” to the tri-axial or omnidirectional accelerometer devices that are worn on the wrist (commonly referred to as wrist actigraphs). Other types of accelerometers used in research such as uniaxial pedometers or accelerometer devices designed to evaluate energy expenditure or activity intensity are fundamentally different from wrist actigraphs and are not discussed here. Despite some technological and operational differences among wrist actigraph manufacturers, the majority of the validated wrist actigraph devices utilize a filter of 0.25 to 2–3 Hz bandpass to ensure that unwanted signals/movements are not recorded and to minimize artifacts resulting from changes in gravitational field [10,24,25]. Detected motions are converted into analog electrical units, which are then digitized and stored as activity units. Generally, activity counts are increased when the acceleration passes the threshold indicated by the filter and usually summarized by time-based epochs. The sampling rate can be programmed and most manufactures provide options for the time-based epochs (e.g., 1 s, 30 s, 1 min, 5 min). The raw activity count data can then be processed in various ways to produce a wide range of variables.

Circadian patterns estimated from actigraphic data are referred to variously as activity rhythms [26], 24 h activity rhythms [27], rest activity rhythms [28], circadian activity rhythms [29,30], and circadian rest-activity rhythms [31]. Here, we collectively refer to the aforementioned methods as behavioral activity rhythms (BAR) since they reflect actual physical behavior rather than biological oscillation identified at cellular levels. To illustrate the approaches discussed in this review, we utilize data from 367 older adults who were enrolled in a large clinical trial evaluating group treatment for co-morbid insomnia and osteoarthritis pain in primary care (The Lifestyles Study, Cognitive Behavioral Therapy for Arthritis Pain and Insomnia in Older Adults, AG031126). This cohort was well-characterized and detailed description on the study sample, design of the trial, and findings were previously reported [32,33,34,35]. The majority of subjects in this cohort completed a baseline actigraphy recording for assessment of sleep. Activity was measured and recorded with the Actiwatch-2 (Phillips/Respironics, Inc., Bend, OR, USA) for seven consecutive days. The Actiwatch-2 weighs 16 g, has a size of 43 × 23 mm × 10 mm, and is equipped with a lithium cell type battery that allows for 91 days of logging time at 1 min epoch lengths as programmed for this study. This accelerometer is a solid-state “Piezo-electric” accelerometer with a range of 0.5–2 G peak value, a bandwidth of 0.35–7.5 Hz, sensitivity of 0.025 G, and a sampling rate of 32 Hz. Some subjects removed the device for extended periods, used it only at nights, or had a malfunctioning device. Those with significant amount of missing data (less than six full days of recordings or having more than 8 h of missing data in a given day) were excluded. A total of 316 participants are included here. For illustration purposes, four individuals (two males and two females) were arbitrarily chosen as examples, and their data were utilized throughout the graphical presentations. 

## 2. Approaches for Estimating Circadian Rhythms from Actigraphy Data 

**Figure 1:**
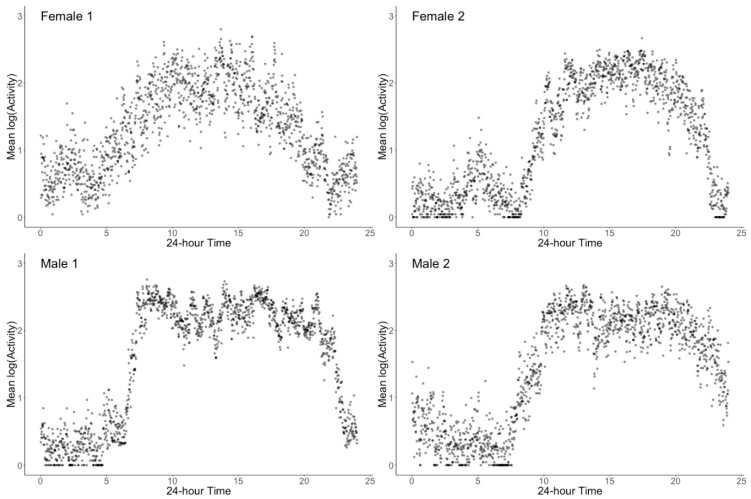
Example illustration of activity patterns of four older adults aggregated from longitudinal actigraphy. Despite individual differences, a repetitive phenomenon is observed that can be described consistently as a period of low activity during the night (sleep), a period of rapid increase in activity in the morning after waking up, variable but markedly higher activity throughout the day, and a final decrease in activity in the evening towards another period of minimal activity.

BAR can be summarized according to an observable behavioral phenomenon with a diurnal pattern having four major characteristics: (1) a period of quiescence (usually sleep), (2) a period of increased activity in the morning following waking up, (3) a relative higher (even though changing) period of wakeful activity during the day, and (4) a period of “winding down” or decreasing activity as the next quiescent period (i.e., sleep) approaches (Figure 1). Parametric [36], non-parametric [37], and a combination of both approaches [28,38] have been used to process the raw actigraphy data and to characterize BAR shape, rhythmicity, robustness, timing, and regularity. 

### 2.1. Parametric Approaches

**Figure 2:**
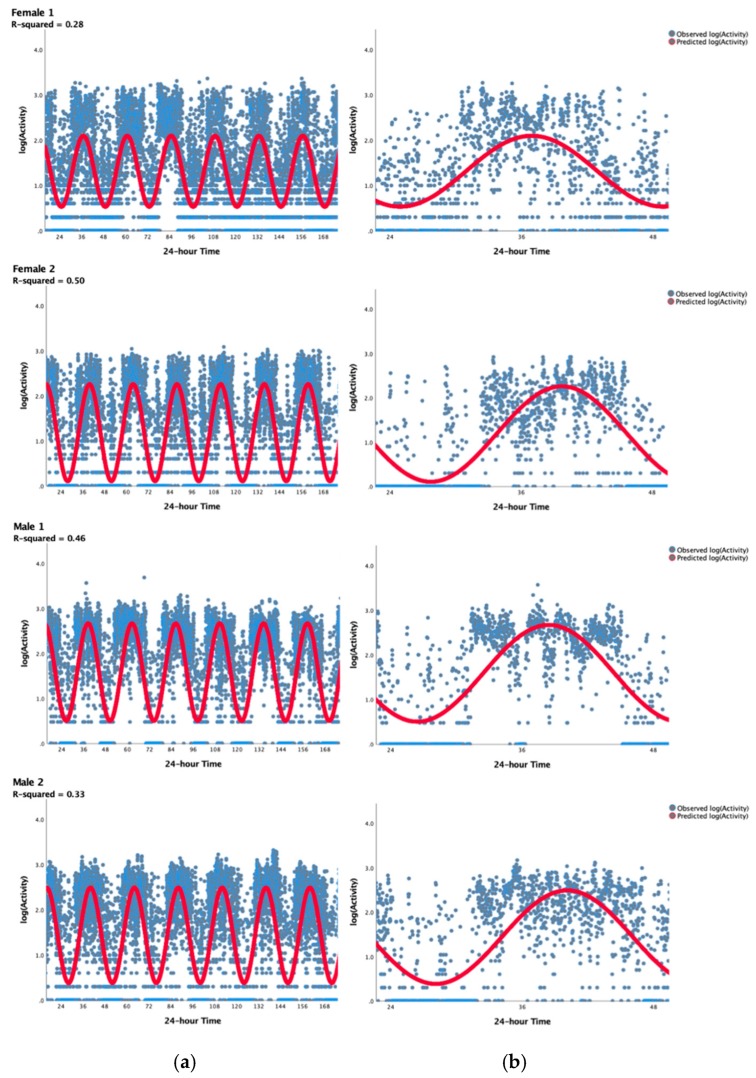
Example illustration of poor fit of cosinor model to observed 7-day activity data of the 4 individuals depicted in Figure 1. (**a**) Figures on the left illustrate the activity rhythm over the entire week whereas (**b**) figures on the right illustrate the overall activity pattern over a single 24 h period. As evident by both figures, the cosinor approach results in extremely poor fit of the model to observed data (mean R^2^ of the entire sample = 0.35, SD = 0.11).

Most commonly, parametric models such as the mathematical cosinor algorithms and Fourier decompositions have been applied to actigraphic data to delineate BAR parameters [5,39]. While most commonly used, cosinor methods have often been criticized for poor model fit (Figure 2) and for the lack of clinical relevance [36,38,40,41]. A glaring example of poor fit is provided by Satlin et al. [41], who used the cosinor approach and reported a “circadian correlation” as a measure of goodness-of-fit of 0.27 in older adults with Alzheimer’s disease (AD) and 0.49 in the healthy controls. In other words, only 7% and 24% of the variance observed in the activity data was explained by a cosinor model. 

The main advantage of the simple cosinor model is that it provides a parsimonious model that requires only three parameters (Table 1). The outcome variables derived from this approach are mesor (midline estimating statistic of rhythm), amplitude (height of the cosine peak), and phi (also called phase or acrophase, the timing of the peak of the rhythm) [26]. Results using this approach tend to focus on amplitude and phi with less emphasis on the mesor. The mesor is the fitted mean of the cosine across the full 24 h including both sleep and wake; this fitted activity average lacks clinical specificity and is rarely reported. The cosinor method uses a parametric approach, meaning it assumes that the data are normally distributed and fit the symmetrical cosinuisoid. The rest/inactive period (i.e., sleep period) within a 24 h day is significantly shorter than the active period; therefore, a symmetrical wave assumption does not properly represent the observed BAR (as in Figure 1) and may be problematic. While highly complex Fourier sequences may partially address this concern and provide better fit, these models quickly lose clinical relevance as they increase within day oscillations and thus are not generally used in clinical sleep and circadian rhythm adult research. Fast Fourier transformations are more commonly used in infants who have expected and multiple sleep bouts during the day [42].

Extended cosine methods have also been introduced [36] and used in various populations including older adults [31]. These extended models are inverse-logit transformations of the standard cosine curve and tend to provide a better model fit with additional parameters but continue to rely on normality assumptions that are fundamentally inaccurate as biological circadian rhythms do not follow such assumptions. Even though extended cosinor approaches provide additional outcome variables as compared to the simple cosinor approach, the majority of studies continue to report only descriptors of model fit, typically amplitude and acrophase. A comprehensive list of the major outcome variables from the extended cosine approach developed by Marler et al. [36] is provided in Table 1. 

### 2.2. Non-Parametric Approches

Non-parametric methods have also been introduced and utilized in older adult populations [37,43,44,45,46]. These models provide several variables including inter-daily stability (IS), intra-daily variability (IV), most active 10 h (M10), least active 5 h (L5), and relative amplitude of the activity pattern (RA; see Table 1 for exact definitions). The main advantage of this approach is that it does not assume normality of the BAR data collected through continuous actigraphic monitoring and early evaluation of such methods demonstrated good discriminative power and improved sensitivity when compared to the cosinor approach [41,44]. However, these outcomes are difficult to interpret clinically as they do not correspond directly to actual observable behavioral patterns. Additionally, this approach does not lend itself to visual characterization of the observable repetitive activity pattern over consecutive days. 

## 3. Characterizing Actigraphy-Derived Behavioral Activity Rhythms in Older Adults

### 3.1. Behavioral Activity Rhythms, Age, and Cognitive Status

BAR tend to weaken with age, becoming more irregular, desynchronized, and attenuated [47]. Older adults tend to have reduced amplitude and rhythmicity (indicated with model fit statistics), as well as worse inter-daily stability and intra-daily variability; however, these results are generally weak and sometimes inconsistent [37,43,46,47,48,49]. Luik et al. [49] evaluated 1734 older adults (average age of 62 ± 9.4 years) and demonstrated that older adults tend to have a more stable BAR, indicating a more repetitive pattern across days, but that older adults also have a significantly more fragmented BAR across the day as they likely have longer sedentary bouts. Disruption in BAR (mainly BAR attenuation) is commonly reported in older adults with dementia [37,41,44,50,51,52]. BAR measures have been shown to be uniquely associated with cognitive performance in older adults without dementia and independent of age effects [53,54]. A large study assessing BAR in 1282 healthy older women reported that decreased amplitude, robustness, and delayed BAR were significant risk factors for the development of mild cognitive impairment and dementia [55]. 

It is very common for patients with dementia to have more activity during the night than during the day, resulting in attenuated BAR [41]. Gehrman et al. [56] carefully evaluated the relationship between BAR parameters and the mini mental state exam (MMSE), a validated and widely used tool for assessing global cognitive functioning, in 188 older adults residing in nursing homes. This study reported a more nuanced relationship where subjects with more impacted rhythms showed lower MMSE scores, but not all subjects with dementia necessarily exhibited BAR disturbances, with the MMSE found not to be related to BAR rhythmicity across the entire sample. In other words, BAR disturbances do not uniformly worsen with progression of dementia processes, suggesting that treatments targeting BAR should be considered independently of the dementia stage with the potential to improve quality of life for these patients and their caregivers [57]. Additionally, some evidence suggests that changes in BAR phase may actually precede cognitive changes in older adults [58]. BAR rhythmicity was shown to be a strong correlate with functional status, well-being, and quality of life in older adults with dementia [54]. Treatments targeting circadian rhythms and directly improving BAR have been studied in different chronic disease populations and tend to result in functional improvement [29]. Yet, most studies are not specific to older adults and the majority of them do not report changes in actigraphic BAR outcomes. Future studies are necessary to establish the effect of interventions targeting actigraphy-derived BAR in older adults with dementia.

### 3.2. Behavioral Activity Rhythms, Mortality, and Quality of Life

Studies have suggested disrupted actigraphy-derived BAR is associated with increased morbidity, mortality, and decreased quality of life in healthy older adults and those with neurodegenerative disorders (e.g., dementia, Parkinson’s disease), yet results are not consistent [30,57,58,59,60,61,62]. BAR disruption has also been commonly reported in chronic diseases, such as cancer and dementia, and is again associated with decreased quality of life independent of age, physical and emotional status [61,62,63,64]. When assessing BAR in 149 older adults with dementia, Gehrman et al. [59] did not find an association between overall BAR rhythmicity and survival rates. However, in a large study including 2964 older men (age 67+) recruited from the general population [60], BAR rhythmicity outcomes were significantly associated with mortality. Participants in the lowest quintile of BAR robustness exhibited a 57% higher mortality rate compared to those with most robust rhythms (i.e., highest quintile), yet the association was not consistent across all the parameters and only present when considering overall rhythmicity using the extended cosine pseudo F-value. No findings were noted when using amplitude or mesor with mortality rate. 

Interestingly, Paudel et al. [60] found the advanced phi/acrophase timing of the BAR showed a 2.8-fold higher rate of cardiovascular disease-related mortality while Gehrman et al. [59] reported a delayed phi/acrophase timing was associated with lower survival rates. In Tranah et al. [30], 3027 community dwelling older women were evaluated for BAR and mortality. While amplitude was associated with all-cause mortality, delayed timing of peak activity (phi/acrophase) was associated with increased mortality from cancer and stroke. Specifically, in this study, a peak BAR of >4:33 PM (approximately, 1.5 SD above the mean) was associated with increased mortality from stroke and cancer. Importantly, the association between BAR abnormalities and mortality was independent of sleep time. While using similar BAR methodology, discrepancies in these studies can be attributed to the utilization of different populations and the use of different methodologies for evaluating mortality and survival. Nonetheless, these results suggest that BAR dysfunction may be a potential preclinical biomarker of mortality and increased risk of serious illness which is independent from normal aging processes. 

### 3.3. Behavioral Activity Rhythms in the Study of Mood in Older adults

Mood disorders are common in older adults and up to 10% of older adults presenting to primary care meet diagnosis of major depressive disorder [65]. The literature linking circadian rhythm disturbances to mood disorders is vast [66,67,68]. Studies have demonstrated an important link between BAR disturbances and depression in older adults [28,54,69]. For example, Smagula et al. [28] showed a negative relationship between BAR robustness and amplitude with depressive symptoms in 2892 community-dwelling older men. Similar findings were reported in Maglione et al. [69] using a cross-sectional sample of 3020 community-dwelling older women (average age of 82.5). Impacted BAR robustness as well as BAR fragmentation seem to be associated with depressive symptoms, yet these associations are minimized when adjusting for covariates of lifestyle and health factors [27,31,70]. While rarely reported, mesor was also a strong predictor of depressive symptoms in older adults [28]. This is expected as reduced activity is commonly shown to be associated with more depressive symptomology [71,72]. 

Research in general adult populations commonly reports a relationship between delayed circadian rhythms and depression [73]. In large studies of older adult women [69] and men [70], non-normal BAR timing was associated with more significant depressive symptoms. However, results are inconsistent and both delayed and advanced BAR have been associated with mood symptoms. Taken together, it seems that timing of the BAR (peak activity time) itself may not be as relevant to mood symptoms as much as increased sedentary time either in the morning or evening [69]. A different study of 238 individuals with a history of mood disorder between the ages of 12 and 90 found that the association between BAR patterns and mood symptoms depended on age, where phase delay was associated with depression in younger age, whereas impacted robustness and disorganized BAR patterns were associated with increased mood symptomology in older adults [48]. 

### 3.4. Methodological Concerns

There is a wide variability in methodology related to actigraphy recording and data management including device placement (dominant vs. non-dominant hand) and epoch length (30 s vs. 60 s are the most common variants). Major differences also include the length of actigraphic recording (number of days) and ways to handle missing data. While battery time varies by manufacturer, most actigraph devices can obtain continuous data for at least 30 days. Nonetheless, few studies have subjects wear an actigraph continuously for this length of time. Of marked importance is the management of missing data. Missing data can occur relatively often and easily in any population, and this is amplified in older adults, especially those with dementia. It is common that research participants remove the device temporarily and forget (or intentionally decline) to place it back. Older actigraph devices were not equipped with off-wrist detection function, though these capabilities are now becoming standard. In older devices, off-wrist times are reported as zeros indicating no activity and this may confound results. The most striking methodological inconsistencies involve the use of various approaches for the estimation of circadian rhythms and reporting of outcomes. Even when studies use similar methods, there are marked discrepancies in reporting of findings and parameters showing no associations are generally not reported at all. Such methodological inconsistencies limit the possibilities of appropriate comparisons between different studies and only allow for an overall discussion of the results. 

## 4. Describing Actigraphy-Derived BAR in Older Adults—A Graphical Approach

When considering which methodology to use in fitting longitudinal circadian/diurnal models for BAR characterization, two highly desirable yet (at times) competing criteria need to be addressed: good data fit and clinically interpretable parameters. The most common criticisms of existing models are either poor model fit (parametric methods) or lack of clinical interpretability (non-parametric approaches). There is an urgent need for models that provide both improved fit and parameters that better characterize the actual observed behavioral pattern. Beyond the statistical and mathematical approaches for summarizing the overall pattern of BAR, studies have been challenged with graphical representations of the activity patterns. Some studies rely on the statistical model to produce visual displays [31,36,48,70], while others use aggregation approaches that summarize longitudinal activity over time [38,41,46,58]. To date, no study has attempted to provide approaches that yield meaningful clinical parameters without sacrificing model fit, relevance, or ability to adequately display and interpret the data. In order to address these significant limitations, our group has developed graphical procedures that can provide both adequate fit of observed data and immediately meaningful clinical outcomes. These suggested methods are not to replace other approaches but rather provide a supplementary technique to organize actigraphy-derived data and improve characterization and estimation of circadian rhythms. 

**Figure 3:**
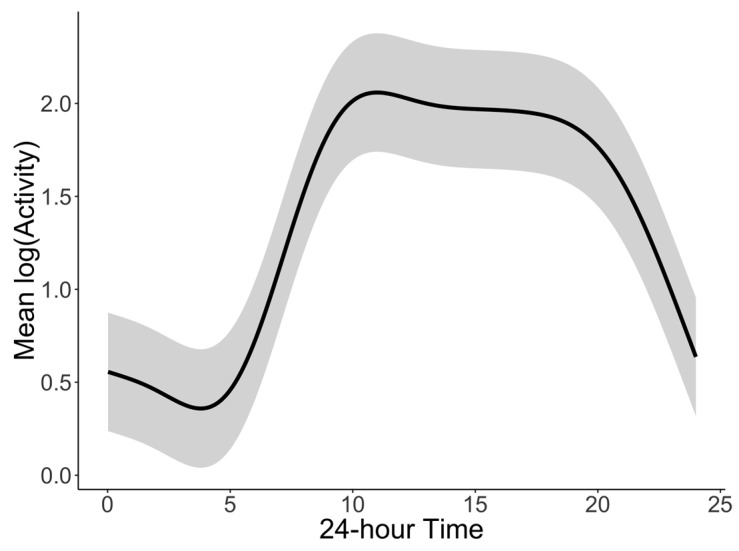
Activity pattern of the entire sample (N = 316) aggregated to a single 24 h period with grey ribbon representing +/−0.5 standard deviation.

The main goal of our approach was to better characterize the observable behavioral phenomenon of BAR that involves four distinct features: (1) a period of sleep and quiescence, (2) a period of increased activity in the morning, (3) a relative plateau period of wakeful activity with some daytime activity changes, and (4) a period of “winding down” or decreasing activity as the next quiescent/sleep period approaches. For this, the accelerometer-derived minute-by-minute activity levels (60 s epoch lengths) were aggregated over seven days for each individual. Similar to previous studies that used this aggregation approach for visualization [38,41,46,58], actigraphy-derived activity can be plotted to summarize diurnal changes in activity with a better face validity. Data aggregation was done using both mean and median; as no differences in results were seen, we present only mean aggregation. As seen in Figure 1, there are marked differences in individual patterns but all follow a similar pattern indicating a period of nighttime inactivity, increase in activity in morning, changing activity pattern during the day with some “dips” of lower activity, and a final decrease of activity at the end of the day until the next period of sleep. Figure 3 shows the aggregation for the entire sample which is greatly smoothed in comparison to the individual plots. This graphical approach of aggregated data (Figure 1) provides a more accurate visual representation of the observable behavioral phenomenon of BAR when visually compared to cosinor approach (Figure 2) using the same individuals. 

**Figure 4:**
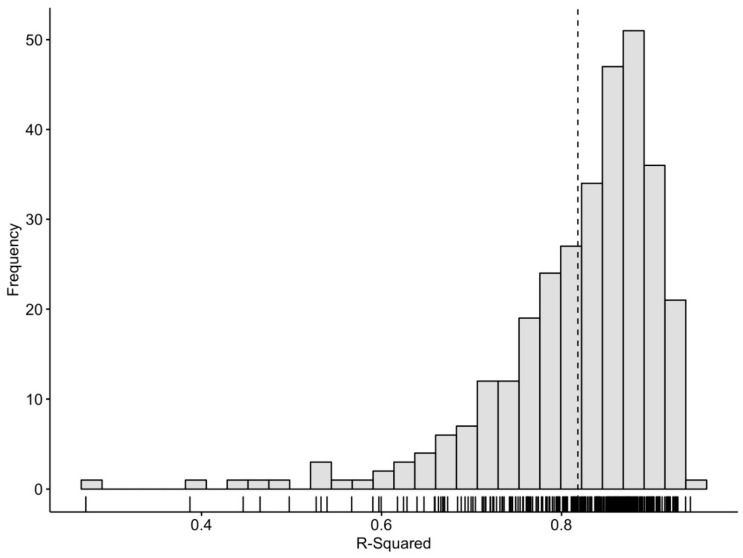
Frequency histogram for R^2^ values from the GAM model for each participant in the sample (n = 316). The dashed line represents the mean R^2^ value for the whole sample (R^2^ = 0.82, SD = 0.09).

**Figure 5:**
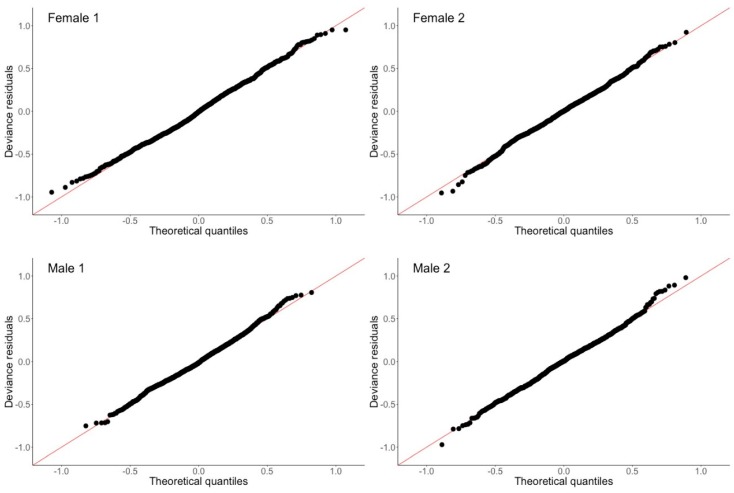
Q-Q plots derived from the GAM for each of the four older adults presented in Figure 6. Each of the plots clearly show that the log-transformed activity data is normally distributed.

The graphical approach we present can be further parametrized. We utilized generalized additive models (GAM) [74] to fit smoothed nonlinear curves to log-transformed aggregated activity measurements with the ‘mgcv’ package [75] for R [76]. GAMs are an extension of the common generalized linear model (GLM) that include smoothed predictors and covariates, in this instance time, in order to model non-linear relationships with the outcome of interest, which here is activity. While not widely used in social and clinical research, GAMs are commonly applied in areas of biological and environmental science for analysis of seasonal and cyclic time-series data, for example in the study of air pollution [77]. As for classic GLMs, GAMs are flexible and may incorporate a number of smoothed or unsmoothed parameters, with a range of approaches available for achieving appropriate smoothing while avoiding overfitting. This is critical in the analysis of actigraphy given the high inter-individual variability of activity profiles and common outliers in activity measurements. Predictors are smoothed using penalized regression splines with smoothing parameters automatically selected by restricted maximum likelihood (REML) during model fitting. This approach avoids over/under-fitting while simultaneously allowing the model to capture individual differences in activity patterns, without requiring the user to have prior knowledge of the form of the distribution. While this automatic selection of smoothing parameters differs from the previously used models for modeling actigraphy data which fit the observed data to the given model (e.g., cosinor), it is key to capturing individual variations in the natural characteristics of the observed activity data. In addition, REML is established as a robust and reliable method for avoiding over/under-fitting [75]. Parameters of individual participant model fits can be examined further with a range of functions included in the ‘mgcv’ package. Assumptions regarding the appropriateness of the data for the GAM can also be easily assessed with standard plots showing the distribution of residuals (Figure 4), as is common practice for GLMs. The Q-Q plots derived from the GAM for each of the four older adults presented in Figure 1 are shown in Figure 5. These plots show that the log-transformed activity data are normally distributed for each of the example participants, as expected.

**Figure 6:**
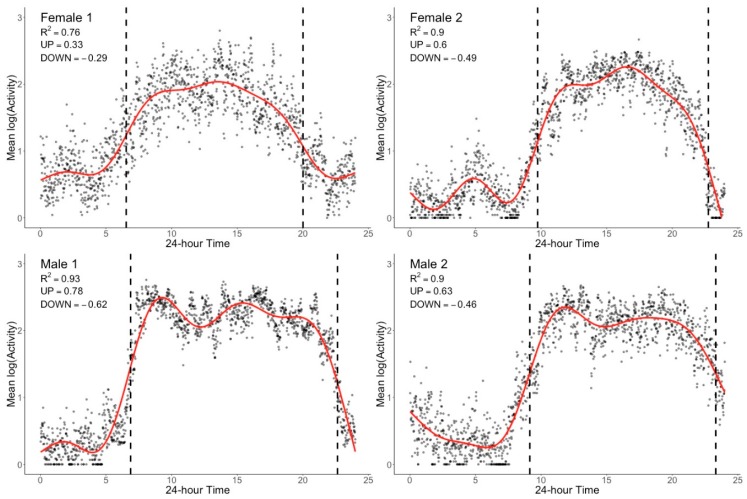
Examples of the smoothed curve (red line) produced by a generalized additive model with aggregated longitudinal actigraphy for the four older adults presented in Figure 1 and Figure 2. R-squared, UP slope and DOWN slope values are presented for each participant, with the dashed lines representing the 24 h time at which the UP and DOWN slopes were calculated (i.e., the time in which the change in activity counts was greatest). It can be seen that the smoothed curves reliably reflect individual changes in activity across the average 24 h period.

There are a range of measures that could theoretically be extracted from the fitted, smoothed curves. Here however, we are interested in capturing the individual characteristics of the onset and offset of activity during the 24 h period, referred to as the UP slope and the DOWN slope, respectively. The points at which the slopes of the UP and DOWN activity periods were at their steepest were calculated from the first derivative of the smoothed curve. From this, the time within the 24 h period of the steepest UP/DOWN slope were also calculated. To ensure complete activity transition periods were captured, even for individuals with delayed or advanced rhythms, the 24 h period used for model fitting was defined as 03:00 to 03:00, with the 24 h period from 00:00 to 24:00 used for plotting data in an intuitive and easily interpretable way. Individual model fits were evaluated by inspecting R^2^ values, the plotted model fit, diagnostic plots of residuals and plots of observed and fitted values. Outcome variables are summarized in Table 2. Of note, the average model fit achieved by this approach (R^2^ = 0.82, SD = 0.09) is markedly higher than that obtained from cosinor approaches (see Figure 6 compared to Figure 2) and indicates that 82% of the variance observed in aggregated mean activity levels are accounted for using this model. Figure 4 shows the distribution of model fit for the entire sample. This negatively skewed distribution indicates that for the wide majority of subjects this model provided relatively high model fit. While this improved model fit is expected as we are modeling aggregated means, these are much more reflective of the observable BAR phenomenon as compared to the cosinor approach using non-aggregated data, and thus result with more meaningful easily interpretable clinical outcomes. 

Figure 6 which presents the same four examples shown in Figure 1 and Figure 2, demonstrates the ability of this approach to meaningfully describe BAR in older adults by capturing individual differences in activity across the 24 h period. Individual differences are immediately noticeable in Figure 6; female 1 had a much slower rate of rising in the morning and winding down in the evening when compared to the other examples provided, resulting in a relatively shorter daytime activity. This participant also had higher activity during the night and lower activity during the day resulting in a lower R^2^ indicating a more attenuated and less rhythmic pattern.

## 5. Conclusions

The utilization of actigraphy in research in older adults has produced significant data that help characterize the behavioral activity rhythms (BAR) of this population and the relevance of BAR to clinical functioning. While methodology and analytic methods vary widely, overall results from the literature have shown that BAR deteriorates with age and becomes more attenuated. BAR has been highly useful in studying clinical adult populations suffering from chronic illness. BAR is more disrupted in patients with dementia, cancer, and pain. There are several limitations for the analytic approaches commonly employed for characterizing BAR and no single method appears to be superior. We found no methods to provide a reasonable model that can depict and represent clearly the observable phenomenon of activity changes during the day. In contrast, we introduced a graphical method that is capable of providing graphic presentation of BAR with high model fit (average of over 80% of variance explained) and distinguishing clinically useful components of BAR. The exclusion of participants with high amounts of missing data has the potential to contribute to the good model fit observed across this sample. However, here, the novel application of GAMs to flexibly capture the natural characteristics of individuals’ BAR resulted in consistently high model fit especially when compared to cosinor approach. Future studies are needed to validate these proposed BAR outcome variables and directly compare these to other methodologies/approaches. Additionally, future studies are required to evaluate the validity of these methods in determining group differences in clinical trials. Researchers should carefully consider the pros and cons of each method and employ that which most closely characterize the component of the rhythm of interest. 

## Figures and Tables

**Table 1:** Circadian outcome variables derived from traditional parametric and non-parametric approaches.

Approach	Variables	Definition	Interpretation
Cosinor model fitting	Midline-estimating statistic of rhythm (mesor)	Mean activity level over the 24 h period	Higher values indicate more average activity across day and night
	Amplitude	Distance between the mean activity level (mesor) and the peak	Higher values indicate higher overall maximum activity amount and more rhythmic changes
	Phi/Acrophase	Time of peak activity in the 24 h period	Later values indicate later peak of activity and may reflects a more delayed phase
	R-Squared	Measure of statistical reliability and consistency of the model-fitted rhythm	Higher values indicate greater robustness of the predicted circadian rhythm
Extended Cosinor Model	Midline-estimating statistic of rhythm (mesor)	Half-way between minimum and maximum	Higher levels indicate more estimated average activity
	Amplitude	Differences between the maximum modelled activity level and the minimum modelled activity level	Higher values indicate higher overall rhythmicity
	Phi/Acrophase	Time of peak activity in the 24 h period	Later values indicate later peak of activity and may reflect a more delayed phase
	Minimum	The lowest point of the fitted curve	Higher values indicate more night-time activity
	Up-mesor	Time from above the mesor to below the mesor	Larger values indicate later time of increasing activity
	Down-mesor	Time from above the mesor to below the mesor	Larger values indicate later time of declining activity
	Alpha	Width of the rhythm	Larger values (wide troughs and narrow peaks) indicates more night time activity
	Beta	Steepness of the rise and fall of the fitted curve	Larger values indicate steeper rise and fall
	R-Squared	Model fit measure	Larger values indicate greater robustness of model fit and more rhythmicity
	F-statistic	An adjustment to the R-Squared while accounting for the number of observations in the model	Larger values indicate greater robustness of the rhythmic pattern and hence overall more rhythmicity
Nonparametric approach	Inter-daily stability (IS)	Invariability of the 24 h rhythm between different days	Higher values indicate better coupling/synchronization of rest-activity rhythm to external zeitgebers (i.e., 24 h cycle)
	Intra-daily variability (IV)	Fragmentation of the 24 h rest-activity rhythm	Higher values indicate increased fragmentation, which may reflect the occurrence of daytime naps and/or nocturnal awakenings
	Daily activity (M10)	Mean activity level during the most active 10 h period of the day	Higher values indicated more active wake period
	Nocturnal activity (L5)	Mean activity level during the least active 5 h period, which usually occurs during sleep and nocturnal arousals	Higher values indicate less restful sleep
	Relative amplitude (RA)	Normalized difference between the most active 10 h period (M10) and least active 5 h period (L5)	Higher values indicate a more robust 24 h rhythm, reflecting higher activity during wake and relatively lower activity during the night

**Table 2:** Proposed outcome variables derived from graphical approach in the lifestyle sample (N = 316).

Measure	Description	Interpretation	Summary Values (Mean, SD, Range)
UP Slope	Slope of fitted curve during period of activity onset where the slope is at its steepest (positive value, Δlog(activity)/hour)	Higher numbers indicate faster (or steeper) increase in activity post awakening.	0.57, 0.16, 0.16–0.98
UP Slope Time	Time within 24 h period at which activity slope is at its steepest (24 h time)	Later values indicate later time of morning increase of activity and may reflect a more delayed awakening time and more delayed phase	07:26, 02:05, 02:59–15:11
DOWN Slope	Slope of fitted curve during period of activity ‘wind-down’ where the slope is at its steepest (negative value, Δlog(activity)/hour)	Higher absolute numbers indicate faster (or steeper) decrease in activity towards the next period of rest.	−0.47, 0.13, −0.12–−0.96
DOWN Slope Time	Time within 24 h period at which activity slope is at its steepest (24 h time)	Later values indicate later time of evening decrease of activity and may reflect a more delayed sleep time and more delayed phase	22:01, 03:52 11:02–05:20
R^2^	Percentage of variance accounted for by model	Larger values indicate greater robustness of model fit and more rhythmicity	0.82, 0.09, 0.28–0.94
